# Phenolic Composition and Antimicrobial Activity of Different Emirati Date (*Phoenix dactylifera* L.) Pits: A Comparative Study

**DOI:** 10.3390/plants8110497

**Published:** 2019-11-12

**Authors:** M. Iftikhar Hussain, Mohammad H. Semreen, Abdallah Shanableh, Muhammad Nasir Khan Khattak, Ismail Saadoun, Islam M. Ahmady, Muath Mousa, Nora Darwish, Wameed Radeef, Sameh S. M. Soliman

**Affiliations:** 1 Research Institute of Science and Engineering (RISE), University of Sharjah, P.O. Box 27272, Sharjah, UAE; shanableh@sharjah.ac.ae; 2 College of Pharmacy, University of Sharjah, P.O. Box 27272, Sharjah, UAE; msemreen@sharjah.ac.ae (M.H.S.); ssoliman@sharjah.ac.ae (S.S.M.S.); 3 Sharjah Institute for Medical Research, University of Sharjah, P.O. Box 27272, Sharjah, UAE; 4 Department of Civil and Environmental Engineering, College of Engineering, University of Sharjah, P.O. Box 27272, Sharjah, UAE; mmousa2@sharjah.ac.ae (M.M.); ndarwish@sharjah.ac.ae (N.D.); wradeef@sharjah.ac.ae (W.R.); 5 Department of Applied Biology, College of Sciences, University of Sharjah, P.O. Box 27272, Sharjah, UAE; mnasir@sharjah.ac.ae (M.N.K.K.); isaadoun@sharjah.ac.ae (I.S.);

**Keywords:** antibacterial activity, antifungal activity, flavonoid content, phenolic content, *Phoenix dactylifera* L.

## Abstract

The biochemical composition, secondary metabolites (phenolic compounds, flavonoids) and antimicrobial potential of different varieties of Emirati date (*Phoenix dactylifera* L.) pits were investigated. Total phenolic acids (TPC) and total flavonoid contents (TFC) of the different date pits were measured using a Folin–Ciocalteau reagent. Different organic solvents [(*n*-hexane; H_2_O: EtOH (1:1); ethyl acetate; acetone: Water (1:1); and methanol: Chloroform (1:1)] were compared to evaluate the phytotoxicity of Ajwa, Fard, Khalas, Khodari, Abu Maan, Lulu, and Mabroom date pits. The antimicrobial activity of the date pit extracts were evaluated by means of agar-well diffusion assay on *Staphylococcus aureus* (ATCC 29123), *Escherichia coli* (ATCC 25922) and *Candida albicans* (ATCC 66027). Minimum inhibitory concentrations (MICs) were measured following clinical laboratory standardization institute (CLSI) protocol. The biochemical analyses of date pits indicate that TPC were ranged from 7.80 mg of equivalent gallic acid⁄100 g dry weight in Ajwa to 4.65 mg in Mabroom. The TFC were ranged between 1.6–4.54 mg of equivalent catechin⁄100 g dry weight. Ajwa pit extract showed good quality traits (higher protein, lower ash content, and intermediate dietary fiber). The results indicate that the ethyl acetate extract of Khalas and Khodari inhibited *S. aureus* with an inhibition zone diameter of 20 mm and MIC of 10 mg/mL. Abu Mann pit extract inhibited the *S. aureus* and also decreased the population of *E. coli*. The diameter of inhibition zone was 15, 16, and 18 mm after treatment with Ajwa extracts, while the MICs were 7.5 and 5 mg/mL. The MeOH: CFM extract of Abu Mann and Ajwa was more potent against *E. coli* bacteria than any other extract. This work demonstrates that the Emirati date pits extract has antimicrobial (antibacterial, antifungal) potential and can be used as phytotoxic natural compounds.

## 1. Introduction

Date palm (*Phoenix dactylifera* L.), is a highly nutrient-rich fruit, originating from the Arabian Peninsula, North Africa, and the Middle East. The worldwide production, utilization and industrialization of dates are increasing continuously [1]. Date fruit is a rich and cheap source of carbohydrates, proteins, amino acids, and minerals (selenium, potassium, calcium, magnesium, manganese, and iron), dietary fiber, vitamins, carotenoids, and fatty acids [2,3]. The biological and therapeutic potential of date flesh and pits demonstrate the presence of polyphenols and flavonoids in both plant tissues [4].

Dates are an important source of different phytochemicals such as flavonoids, phenolic acids, anthocyanins, tannins, and carotenoids [5,6]. Phenolic compounds, volatile compounds, flavonoids (apigenin, luteolin, and quercetin), phytosterols (*β*- sitosterol, iso fucosterol, stigmasterol, campesterol), and carotenoids (lutein, neoxanthin, *β*-carotene, violoxanthin, antheraxanthin) were also reported in date fruits and pit extracts [7,8]. Other authors also documented different polyphenols such as, *p*-hydroxybenzoic acid, gallic acid, protocatechic acid, vanillic acid, *o*-coumaric acid, caffeic acid, syringic acid, ferulic acid, *p*-coumaric acid, 3-caffeoylquinic acid, and 3-*O*-caffeoylshikimic acid [9]. Different date varieties contained soluble phenolic compounds like hydroxybenzoates, hydroxycinnamates and flavonols [10]. The ripe stage of Khasab, Fard, and Khalas were reported to contain 20, 35, and 63 mg/100 g of phenolic acid, respectively with ferulic acid being the dominant [11]. Some clinicians also elaborated the low incidence of diabetes, cancer, heart and lung diseases due to judicious use of fruits and vegetables [4,5,6,8,12]. Therefore, in developing countries, the use of plant-derived natural products and herbal medicines has significantly increased as alternative solutions to health problems. In this context, the screening, selection and evaluation of pharmacological properties of plant-based natural phytochemicals should be included in the alternative form of health care.

The seeds, stones, and pits of several fruits are used as complementary medicine because of their phytochemical nature that helps to prevent sickness, cure the disease, reduce the side effects, and different kind of stresses. Several reports on date pits showed their functional food properties in dietary treatment [13], macro- and micronutrients [14], phenolic acids [2], as a bread ingredient [15], as well as protein solubility [16]. These investigations demonstrate that date pits are not a waste product but a bulk source of natural antioxidants. Antibacterial prospects of various date pits were also reported by various authors against Gram-negative and positive bacteria [17,18,19] and fungi. Furthermore, it was reported that the extraction solvent plays a significant role in determining the phenolic content and these contents are always higher in aqueous extracts in comparison to alcoholic extracts [20,21]. The main objective was to evaluate the effect of date pit extracts on Gram-positive (*S. aureus*), Gram-negative (*E. coli*), bacterial pathogens, and fungi (*Candida albicans*). Meanwhile, the total phenolic compounds and flavonoids will be identified from different date pits in order to develop which kind of secondary metabolites are present within the pits and their potential phytotoxicity.

## 2. Materials and Methods

### 2.1. Date Pits Material

The fruits of date palm varieties, Abu Maan, Fard, Khalas, Ajwa, Mabroom, Lulu, and Khodari were purchased from Al FOAH COMPANY, Al Saad, Al Ain, Abu Dhabi, United Arab Emirates (UAE). Ripe fruits of best quality, and free from fungal and pest damage were used for this study. Information regarding the cultivars and origin were known based on the information supplied by the supplier. The fruit samples were transported to laboratory and saved at 4 °C until analyzed.

### 2.2. Date Fruit De-Pitting and Collection of Pits

The pits were removed using a sharp knife at Environmental Engineering lab, University of Sharjah, UAE. The pits were washed with distilled and deionized water and dried at room temperature using two layers of filter papers. The dried pits were crushed and grinded using a grinder (Knifetec 1095 laboratory mill) (10,000 rpm) to make fine powder.

### 2.3. Organic Solvent Extraction and Treatment Solution Preparation

Different organic solvents, hexane, H_2_O: Ethanol (EtOH) (1:1), acetone: Water (1:1), ethyl acetate, MeOH: Chloroform (1:1), were used to obtain extracts from different date pits. The date pit powder (20 g) was soaked in selected organic solvent (600 mL) and stirred gently for 24 h at 25 °C. The ingredients attained equilibrium in the organic solvent and then the solvent extract was centrifuged and the supernatant was evaporated to dryness in rotary-evaporators under vacuum at 50 °C. The dry residue was collected, weighed, and stored in air sealed containers at −4 °C until use. The treatment solution (10 mg/mL) was prepared for each pit extract using dimethyl sulfoxide (DMSO). All the treatment solutions were prepared in sterilized conditions by filtering the treatment solutions through Millipore filters (0.45 μm) and stored in the lab fridge at 4 °C. The initial concentration (10 mg/mL) used for testing the antimicrobial activities was further aseptically diluted to determine the minimum inhibitory concentrations (MIC) of the extracts.

### 2.4. Proximate Analysis

The moisture contents were measured by vacuum oven (method 934.06), protein by Kjeldahl nitrogen (method 920.152), and ash by direct analysis (method 940.26) as per guidelines of the Association of Official Analytical Chemists methods [22]. The crude protein percentage was measured through the total nitrogen content and multiplied by a factor of 6.25. The lipid contents were evaluated by the method of Bligh and Dyer as described by Hanson and Olley [23]. Total carbohydrates were calculated as reported previously [24].

### 2.5. Dietary Fiber Analysis

The dietary fiber was measured using the AOAC enzymatic gravimetric method 991.43 [22].

### 2.6. Total Phenolic Content (Folin–Ciocalteau Assay)

The Folin–Ciocalteau reagent (FCR) is used for determination of total phenolic contents [25]. The pit extract (40 μL) was thoroughly mixed with FCR (1.8 mL) and left in the lab at room temperature for five min. In this mixture, the sodium bicarbonate (7.5%; 1.2 mL) was added and samples were kept at 24 °C for 60 min. The absorbance was measured later using spectrophotometer at 765 nm and results were expressed as mg gallic acid equivalent (GAE)/100 g sample [26]. Gallic acid was used as standard in these measurements.

### 2.7. Total Flavonoids

The flavonoids were determined according to procedure described by Kim et al. [27]. In the pit extract (1 mL), distilled water (4 mL) was added followed by 5% sodium nitrite solution (0.3 mL) mixing and 10% aluminum chloride solution (0.3 mL). The test tube containing the mixture was incubated for 5 min at ambient temperature and 2 mL NaOH (1 M) was added. Distilled water was supplemented to make the volume 10 mL. The sample was mixed by using vortex and the pink color absorbance was measured at 510 nm. A calibration curve was prepared with catechin and the results were expressed as mg catechin equivalents (CEQ)/100 g sample.

### 2.8. In Vitro Anti-Microbial Bioassays

The (ATCC) microbial strains were obtained from the stock culture available at the Department of Applied Biology, University of Sharjah, UAE. Three experimental studies were carried out with the main objectives (i) to assess the antimicrobial properties of two popular date varieties (Khalas, Khodari) pit extracts using three organic solvents (hexane, ethyl acetate, H_2_O: EtOH (1:1) (treated and untreated with UV) on American type culture microbial strains (ATCC), that is, Gram-positive, *Staphylococcus aureus* (ATCC 29213), Gram-negative, *Escherichia coli* (ATCC 25922) bacteria and the fungus, *Candida albicans* (ATCC 66027); (ii) to identify the main phenolic compounds and flavonoids present in the six date palm (Abu Maan, Fard, Khalas, Ajwa, Mabroom, Lulu) pit extracts, (iii) and to evaluate the antibacterial and antifungal activity of these date pit extracts using three different organic solvents (acetone + water (1:1), ethyl acetate, and MeOH + chloroform (1:1).

The antimicrobial activity of pit extract of each variety was first evaluated by the agar ‘well diffusion method’ according to Clinical and Laboratory Standards Institute protocol (CLSI) [28,29]. The microorganisms were grown in nutrient agar overnight at 37 °C. A standard suspension with turbidity equal to 0.5 McFarland was prepared on sterile normal saline to give bacterial concentration of 1–2 × 10^8^ CFU/mL. Sterile cotton swabs were dipped in the appropriate test organism suspension and evenly streaked over the entire surface of agar plate to obtain semi confluent growths. Six wells (6 mm) per plate were made with the reverse side of the sterilized micropipette tips. Each well was filled with 50 μL of the respective filter-sterilized pit extract using a micropipette. DMSO was used as the negative control. The experiment was conducted under sterilized conditions in a laminar air flow cabinet and then the plates were incubated at 37 °C for 24 h. Standard antibiotics such as amikacin (30 µg) was used as a positive control for *S. aureus* and *E. coli* and fluconazole (25 µg) was used for *C. albicans*. Each extract was analyzed in triplicate. The presence of inhibition zones was observed and their size was measured in mm [28,29].

### 2.9. Minimum Inhibitory Concentration

The MIC is lowest concentration of a antimicrobial agent that can inhibit the visible growth (turbidity) of test microorganisms after overnight incubation. The MICs was evaluated for all date pit extracts using both the micro dilution method and the spectrophotometric assay according to CLSI [28,29].

### 2.10. Statistical Analysis

General Linear Models (GLM) procedure of SPSS software (version 24.0) for Windows (SPSS Inc., Chicago, IL, USA) was used to perform analysis of variance (ANOVA). Data were presented as mean ± standard error and differences among treatment means were compared using Tukey’s HSD test at *P* < 0.05 significance level.

## 3. Results

### 3.1. Proximate Analysis

Table 1 shows the biochemical evaluation of the selected six date varieties pits that includes moisture, fats, protein, ash, and carbohydrates levels. There was a significant difference between moisture contents that ranged between 9.72–16.28%. The Khalas variety possesses the highest moisture content, while the Fard variety had the lowest moisture level. The fat content ranged between 8.25–12.57%. Mabroom showed higher fat content, whereas Abu Maan had a lower fat content. There was significant difference in protein levels that ranged between 5.21–8.62%, and the Ajwa variety showed the highest while Khalas possessed the lowest protein content (Table 1). The ash content ranged between 0.15–1.80%. Khalas had the lowest ash content, whereas Abu Maan had the highest ash content. There was also a significant difference in carbohydrate contents that ranged between 2.1–4.8%. The Khalas pits had the maximum while Mabroom had the lowest carbohydrate content. The difference in the biochemical traits of date pits was due to different growth, agronomical, cultural practices, harvest time variation, and post-harvest processing conditions.

### 3.2. Dietary Fiber Composition

There was significant difference in dietary fiber content (D.F.) among date varieties pits that ranged between 68.04–75.5% (Table 1). DF was lowest in Mabroom, whereas the highest was in Abu Maan.

### 3.3. Phenolic Compounds and Flavonoids

The phenolic compounds were highest in Ajwa (7.80 mg GAE/100 g dw) followed by Khalas (7.28 mg GAE/100 g dw), while they were the lowest in Mabroom (4.65) mg gallic acid equivalents/100 g dry weight (Table 2). The total flavonoid contents varied significantly and the highest flavonoid contents were found in Ajwa (4.54) followed by Khalas (4.3), whereas lowest in Fard (1.6) mg in terms of catechin equivalents/100 g DW of sample (Table 2). The difference between flavonoids among different date varieties pits was also significant.

### 3.4. Experiment I

The results in Table 3 clearly indicate that the ethyl acetate extract (untreated UV) of the popular date palm pits (Khalas and Khodari) inhibited *S. aureus* with a 20 mm inhibition zone. The ethyl acetate extract of Khalas pits inhibited *E. coli* (15 mm inhibition zone). However, Khalas and Khodari pits extracted with either hexane or (H_2_O: EtOH (1:1) exhibited no activity against any of the tested organisms (Table 3). None of the pits extracted with either ethyl acetate or hexane or (H_2_O: EtOH (1:1) showed any phytotoxic activity against *C. albicans*.

Table 4 describes the activity of date pit extracts (UV treated samples) against different bacteria and *C. albicans*. UV-treated ethyl acetate extracts of Khalas and Khodari date palm pits showed phytotoxicity against *S. aureus* (20–22 mm inhibition zone) (Table 4). However, the ethyl acetate extracts of Khodari date palm seeds revealed an activity against all tested pathogens except *C. albicans* and with inhibition zones ranged between 11 and 22 mm. The data indicated that Khodari date pits extracted with ethyl acetate or hexane inhibited the tested bacteria but not *C. albicans* (Table 4).

### 3.5. Experiment II

The acetone: Water (1:1) extract of Khalas variety showed significant antimicrobial activity against *S. aureus*. The acetone: H_2_O and ethyl acetate extract of Khalas pits inhibited Gram-negative bacteria (*E. coli*) (Table 5).

Similarly, acetone: H_2_O (1:1) extracts of Abu Mann and Ajwa date pits significantly inhibited *S. aureus*. Meanwhile, the acetone: H_2_O (1:1) extracts of the same varieties also decreased the population of *E. coli*. The diameter of the inhibition zone was 15, 16, and 18 mm following treatment with Ajwa date pit extracts (10 mg/mL) from all the three organic solvents, acetone: H_2_O (1:1); ethyl acetate: ETAC, and MeOH: Chloroform (1:1): MeOH: CFM) (Table 5). We also found that the MeOH: CFM extract of Abu Mann was more potent against *E. coli* bacteria than acetone and ethyl acetate extract. In both cases, the diameter of inhibition zone was 15 mm. All the organic extracts from Ajwa variety also exhibited toxic impact on *S. aureus* bacterial colony. However, the MeOH: CFM extract of Ajwa was more lethal that significantly inhibited the *E. coli* bacteria and diameter of inhibition zone was greater (18 mm) than rest of the treatments (Table 5).

The methanolic, ethyl acetate and acetone extracts of Fard and Mabroom varieties pits did not show significant phytotoxicity without any inhibition zone in *E. coli*. The antimicrobial properties of natural product largely depend upon solvent, organism tested, and plant part used. The present results showed that date pit extract possesses antibacterial activity against the tested organisms; however, pits extracted with MeOH: CFM solvents were more effective antimicrobial agents than the other two extracts (Table 5). Across all the extracts from different varieties exhibited, the values corresponding to bacterial inhibition zones produced by the Khodari (ethyl acetate) (22 mm), Khalas (ethyl acetate) (20 mm), and Ajwa (methanol: Chloroform) (18 mm) were close to the inhibition zones of standard antibiotics (amikacin) indicating the efficacy of natural products. DMSO (negative control) did not show any activity against the bacterial strains (Table 5).

The MeOH: CFM extract of Mabroom and acetone: H_2_O extract of Ajwa showed similar antibacterial activity against *S. aureus* (inhibition zone diameter, 13 mm). The phytotoxicity of different extracts from Lulu was quite different than the other date palm varieties. The acetone: H_2_O extracts (10 mg/mL) of Lulu significantly inhibited the *S. aureus* and *E. coli* and diameter of inhibitory zone was 10 mm and 12 mm, respectively (Table 5).

### 3.6. Minimum Inhibitory Concentration

The MICs of different date pit extracts against the *S. aureus* (ATCC 29213), *E. coli* (ATCC 25922) lies between 2.5 and 10 mg/mL (Table 6).

## 4. Discussion

Dietary fiber (D.F.) varies between date pits and was in the range of 68.04–75.5% while the highest DF was observed in Abu Maan and the lowest in Mabroom. Dietary fibers are comprised of different plant-based substances that escape the digestion during their transit through the upper gastrointestinal tract [30,31]. The total DF in different date varieties ranges from 6.5–11.5% which shows variation with respect to variety and climate [32]. The polysaccharides in dates comprised of *β*-glucan, arabinoxylans, and cellulose. DF has important therapeutic benefits such as anti-diabetic and anti-obesity effects and exhibits a protective effect to maintain a good gut health [33,34]. Moreover, important functional properties which are needed for application in the food industry, for example, water-holding, oil-holding, and emulsifying and/or gel formation have also been demonstrated by DF. The desirable properties and stable emulsion-based food products can be developed by incorporating DF in various food products. Mrabet et al., [35] attempted to extract and analyze dietary fiber from Tunisian secondary date fruit varieties and reported that the content of dietary fiber ranged between 4.7 and 7 g/100 g, thus giving strong indication that the date DF can be utilized as additives in fiber-enriched food [36]. Furthermore, the dietary fiber is well-known for absorption of cholesterol and declines its availability to the human body and thus renders many health benefits including preventing cardiovascular diseases. Furthermore, the insoluble fraction of the dietary fibers provides a bulking effect and helps in the generation of short chain fatty acids in the intestine during the fermentation [37]. Health and nutrition specialists agree on the daily consumption of DF, but a recommended daily intake has not been set. However, a daily intake of 25 g/day has been established by well-known nutrition experts. Meanwhile, soluble and insoluble fibers possess different health benefits and consequently more research will further enhance the current knowledge about the quantity and quality profiles of date pits.

Ajwa showed the highest phenolic compounds followed by Khalas. Similarly, Mansouri et al. [38], also reported a similar quantity of phenolic compounds in Algerian date fruits, except for Kharak date fruits that was significantly higher than for the rest of the varieties. Wu et al. [39] documented the phenolic compounds in the range of 572–661 mg gallic acid equivalents/00 g fresh weight. Ajwa dates were also analyzed for the total phenolic content which varied from 245–455 mg/100 g. However, it was reported that the extraction procedure and organic solvent might interfere in the extraction of polyphenols. Other researchers documented that the contents are always higher in aqueous extract in comparison to alcoholic extracts [20,21]. Various levels of polyphenols like, *p*-hydroxybenzoic acid, gallic acid, protocatechic acid, vanillic acid, *o*-coumaric acid, caffeic acid, syringic acid, ferulic acid, *p*-coumaric acid, 3-caffeoylquinic acid, and 3-*O*-caffeoylshikimic acid have been identified in dates [9]. Different date varieties contained phytochemicals like hydroxybenzoates, hydroxycinnamates, and flavonols [10]. The ripe stages of Khasab, Fard, and Khalas were reported to contain 20, 35 and 63 mg/100 g of phenolic acid, respectively [11]. The 5-*O*-caffeoylshikimic acid and its isomers like isodactyliferic acid and neodactyliferic acid were reported from Algerian dates (Deglet Noor) [38].

A similar pattern of variation was found regarding flavonoid contents that were the highest in Ajwa and the lowest in Fard (Table 2). The difference between flavonoids among different date varieties pits was significant. It was reported that Ajwa contain rutin (0.65–0.85 mg/100 g), catechin (0.73 mg/100 g), and caffeic acid (0.57–1.84 mg/100) [20,21]. The phenolic content of Ajwa varied according to their ripening stage (10 mg/100 g–290 mg/100 g). The high polyphenols content is reported in Ajwa at the kimri stage (290 mg/100 g) followed by khalal (150 mg/100 g), rutab (20 mg/100 g) and lowest amount was found in the tamr (10 mg/100 g) stage [6]. Date fruit displays considerable decrease in polyphenols with the advancement of ripening and increase in its concentration is only seen when the fruit underwent physical damage and microbial infection [40].

Exploring the antibacterial activities of date pits, several authors also reported the presence of polyphenols, alkaloids, flavonoids, tannins, and steroids that work towards the combined action as antioxidants. These secondary metabolites can serve as potent natural products and phytochemicals and significantly retard the microbial growth, proliferation and infection [41]. These compounds may act individually as a biologically active compound or provide a synergistic effect to achieve antibacterial properties [41]. Similarly, in another study, the methanolic extract of Ajwa had antibacterial activity against *E. coli*, *Bacillus cereus*, *S. aureus*, and *Serratia marcescens* [42]. The antibacterial, antifungal and anti-viral activity of extracts obtained from medicinal plants normally depends upon organic solvent, target organism, and plant parts used. Shakiba et al. reported that among all the pits extract tested, the MeOH: CFM extract was more potent at inhibiting the microbial activity. There was no inhibition zone observed for *E. coli* (PTCC 1270), *E. coli* (PTCC 1399), and *S. marcescens* for the methanol extract of Mazafati. However, the same extract inhibited the *E. coli* (PTCC 1330) [43]. This shows that the antibacterial properties of the methanolic extract of the same date palm variety could also possibly be selective against certain strains of bacteria [43]. Khatami et al. [44] reported that date pit aqueous extract treated with silver nanoparticles (AgNPs) decreased the *Rhizoctonia solani* (AG2_2) population at 25 µg/mL while it also significantly reduced the *Klebsiella pneumonia* (PCI 602), and *Acinetobacter baumannii* (ATCC 19606). In another study, the acetone and methanol extracts of three date pits showed antibacterial activity against *Bacillus subtilis, Escherichia coli, Pseudomonas aeruginosa, Shigella flexeneri, Staphylococcus aureus*, and *Streptococcus pyogenes,.* However, aqueous extract had poor phytotoxic impact against all tested microbial pathogens and a negligible effect on *P. aeruginosa*. Meanwhile, pits extracts were found to be more effective than leaf extracts [18]. Earlier, it was reported that methanol and acetone extracts of the *P. dactylifera* pits moderately inhibited the growth of Gram-positive and Gram-negative bacteria [45]. Another study conducted in Iran on four different date pits demonstrated that all pit extracts showed inhibitory effect against *Staphylococcus aureus*, but not against *Escherichia coli.* Minimum inhibitory concentration and minimum bactericidal concentration of the extracts ranged from 1.56–3.125 and 3.125–12.5 mg mL^−1^ for *Staphylococcus aureus,* respectively [46]. It was reported that the phytotoxicity of date pits is mainly due to the presence of secondary metabolites such as cinnamic acids, flavonoid glycosides and flavanols [44,45,46].

Several researchers documented that the date fruit possesses strong antiviral, antibacterial, and antifungal potential [45]. The ethanol and water extracts of Egyptian date fruits had strong antimicrobial activity against five pathogenic bacterial strains and it was found that the water extracts of date fruit had strong antimicrobial potential against diarrhea-causing bacteria *Salmonella* ssp. and *Shigella* spp. [47]. While studying the antifungal activity of aqueous date fruit extract with amphotericin B (antifungal drug), it was found that the therapeutic index of amphotericin B increased significantly when used together with the extract. Moreover, the cytotoxicity (of red blood cells) otherwise induced by amphotericin B was also prevented by the aqueous date extract [48]. Natural compounds such as phenolic acids, flavonoids, tannins, alkaloids, and steroids present in the plant extract are mostly responsible for herbicidal [49,50,51], antifungal [52], and antimicrobial activities [42]. The natural toxins might act individually as pharmaceutically active compounds or provide synergistic effect to exhibit the antimicrobial properties [42]. The phytotoxicity of phenolic compounds depends upon the presence of hydroxyl group (number and location) [53]. Flavonoids also exhibited antimicrobial potential and their mode of action was routed through reduction in activity of nucleic acid synthesis, disturbance in energy metabolism and cytoplasmic membrane functions [42,54]. The present study indicates that the extraction of secondary metabolites depends upon the solubility and polarity of organic solvents. It was found that methanol and chloroform could extract the phenolic compounds and flavonoids from date palm pits more than other solvents, and acetone alone did not perform very well. However, the efficiency of organic solvents also depends on the plant species [55]. However, other researchers have diversified opinions and they documented an aqueous extract, methanol, and acetone for phenolic extraction that varies from each other [56,57,58].

## 5. Conclusions

We concluded that pits constitute large quantities of fiber that have potential health benefits. Quantitative screening of selected date (Ajwa, Fard, Khalas, Abu Maan, Lulu, and Mabroom) pit extracts showed the presence of a significant amount of secondary metabolites (SM) (phenolic compounds and flavonoids). Date pits extracted from Ajwa, Abu Maan and Khalas using different organic solvents (acetone + water and MeOH + chloroform) exhibited a large quantity of phytochemicals, antimicrobial properties and other quality attributes. Ajwa, Khalas, and Khodari revealed to be potent against bacterial pathogens. Different date varieties showed different nutritional, health and phytochemical properties and can be used in everyday life as a natural source of nutrient and therapeutic purposes.

## Figures and Tables

**Table 1:** Biochemical composition (proximate analysis and dietary fiber) of six date palm pits.

Date Palm Variety	Moisture	Fats	Protein	Ash	Carbohydrates	Dietary Fiber
Khalas	16.28 ± 0.25 a	10.24 ± 0.04 b	5.21 ± 0.06 c	0.15 ± 0.12 b	4.80 ± 0.06 a	72.74 ± 0.10 c
Lulu	11.14 ± 0.06 c	8.45 ± 0.22 c	6.25 ± 0.10 b	1.45 ± 0.05 a	3.20 ± 0.14 b	70.10 ± 0.20 d
Fard	9.72 ± 0.20 e	10.72 ± 0.10 b	5.74 ± 0.14 c	0.50 ± 0.22 b	3.40 ± 0.10 b	70.25 ± 0.24 d
Ajwa	10.6 ± 0.04 d	10.45 ± 0.06 b	8.62 ± 0.06 a	0.74 ± 0.10 b	2.80 ± 0.10 c	74.60 ± 0.18 b
Abu Maan	10.32 ± 0.14 d	8.25 ± 0.04 c	5.67 ± 0.14 c	1.80 ± 0.26 a	3.20 ± 0.20 b	75.50 ± 0.13 a
Mabroom	12.44 ± 0.10 b	12.57 ± 0.10 a	6.2 ± 0.20 b	1.24 ± 0.04 a	2.10 ± 0.22 c	68.04 ± 0.15 e

Data are expressed as mean ± S.D. (*n* = 3) on a dry weight basis. Means ± SD followed by the same letter, within a column, are not significantly different (*p* > 0.05).

**Table 2:** Total phenolic contents and total flavonoid in six date palm varieties pits (dry weight basis).

Date Palm Variety	Total Phenolic Content (mg GAE/100 g dw)	Total Flavonoid Contents (mg CEQ/100 g dw)
Khalas	7.28 ± 0.02 a	4.30 ± 0.02 a
Lulu	6.40 ± 0.03 b	2.70 ± 0.04 c
Fard	6.10 ± 0.020 b	1.60 ± 0.01 d
Ajwa	7.80 ± 0.05 a	4.54 ± 0.0 a
Abu Maan	5.00 ± 0.01 c	3.80 ± 0.10 b
Mabroom	4.65 ± 0.04 c	3.50 ± 0.04 b

Values are the mean ± standard error of three samples. Means ± SD followed by the same letter, within a column, are not significantly different (*p* > 0.05). GAE, gallic acid equivalents. CEQ, catechin equivalents.

**Table 3:** Activity of un-treated date palm pit extracts against different microbial strains (bacteria and fungi).

#	Extract	Inhibition Zone Diameter (mm)		
		*Staphylococcus aureus* (ATCC29213)	*Escherichia coli* (ATCC 25922)	*Candida albicans* (ATCC 66027)
1	Khalas (Ethyl acetate)	20 ± 1.2 a	15 ± 1.1	0
2	Khalas (Hexane)	0	0	0
3	Khalas (H_2_O: EtOH (1:1)	0	0	0
4	Khodari (Ethyl acetate)	20 ± 1.3 a	0	0
5	Khodari (Hexane)	0	0	0
6	Khodari (H_2_O: EtOH (1:1)	0	0	0

Values are the mean ± standard error of three samples. The mean with different letters in a column differed significantly at *p* < 0.05. For standard amikacin (30 μg) was used for *S. aureus* and *E. coli* and inhibition zone size (22 mm and 25 mm respectively); fluconazole (25 μg) used for *C. albicans* (29 mm).

**Table 4:** Activity of UV-treated date palm pit extracts against different microbial strains (bacteria and fungi).

#	Extract	Inhibition Zone Diameter (mm)		
		*Staphylococcus aureus* (ATCC29213)	*Escherichia coli* (ATCC 25922)	*Candida albicans* (ATCC 66027)
1	Khalas (ethyl acetate)	20 ± 1.0 b	0	0
2	Khalas (hexane)	0	7 ± 0.45 b	0
3	Khalas (H_2_O: EtOH (1:1)	12 ± 1.0 d	7 ± 0.6 b	0
4	Khodari (ethyl acetate)	22 ± 1.5 a	11 ± 0.9 a	0
5	Khodari (hexane)	17 ± 1.2 c	12 ± 1.0 a	0
6	Khodari (H_2_O: EtOH (1:1)	0	0	0
7	Amikacin (30 μg)	22	25	-
8	Fluconazole (25 μg)	-	-	29

Values are the mean ± standard error of three samples. The mean with different letters in a column differed significantly at *p* < 0.05. For standard amikacin (30 μg) was used for *S. aureus* and *E. coli* and inhibition zone size (22 mm and 25 mm respectively); fluconazole (25 μg) used for *C. albicans* (29 mm).

**Table 5:** Activity of date palm pit extracts against different microbial strains (bacteria and fungi).

	Bacteria	Treatment	Diameter of Inhibition Zone (mm)					
			Khalas	Abu Maan	Ajwa	Fard	Lulu	Mabroom
Gram-positive		Acet + H_2_O	13 ± 0.3 a	15 ± 0.3 a	13 ± 0.2 a	6 ± 0.1 b	10 ± 0.2 a	6 ± 0.1 b
	*S. aureus*	ETAC	8 ± 0.2 c	8 ± 0.19 b	11 ± 0.19 b	6 ± 0.1 b	6 ± 0.1 b	6 ± 0.1 b
	(ATCC29213)	MeOH + CFM	9 ± 0.2 b	15 ± 0.4 a	10 ± 0.2 c	10 ± 0.2 a	6 ± 0.1 b	13 ± 0.3 a
Gram-negative		Acet + H_2_O	13 ± 0.3 a	12 ± 0.3 b	15 ± 0.4 c	6 ± 0.19 a	12 ± 0.3 a	6 ± 0.1 a
	*E. coli*	ETAC	12 ± 0.3 b	12 ± 0.19 b	16 ± 0.38 b	6 ± 0.18 a	10 ± 0.19 b	6 ± 0.14 a
	(ATCC 25922)	MeOH + CFM	10 ± 0.2 c	15 ± 0.34 a	18 ± 0.5 a	6 ± 0.2 a	6 ± 0.17 c	6 ± 0.12 a
Fungi		Acet + H_2_O	0	0	0	0	0	0
	*C. albicans*	ETAC	0	0	0	0	0	0
	(ATCC 66027)	MeOH + CFM	0	0	0	0	0	0

Acet + H_2_O: Acetone + water (1:1); ETAC: Ethyl acetate; MeOH + CFM: MeOH + chloroform (1:1); Treatment: Date palm pit extracts: 10 mg/mL. Values are the mean ± standard error of three samples. The mean with different letters in a column differed significantly at *p* < 0.05. For standard amikacin (30 μg) was used for *S. aureus* and *E. coli* and inhibition zone size (22 mm and 25 mm respectively); fluconazole (25 μg) used for *C. albicans* (29 mm).

**Table 6:** Minimum inhibitory concentration (MIC) of date palm pit extracts against American Type Culture bacterial strains, *Staphylococcus aureus* and *Escherichia coli*.

Code	Date Palm Variety	Organic Solvents	Date Pit Extract (mg/mL)
			(MIC)
A1	Khalas	Acetone + water (1:1)	10 ± 0.3
B1	Abu Maan	Acetone + water (1:1)	10 ± 0.3
B3	Abu Maan	MeOH + chloroform (1:1)	2.5 ± 0.3
C1	Ajwa	Acetone + water (1:1)	7.5 ± 0.5
C3	Ajwa	MeOH + chloroform (1:1)	5 ± 0.3
D3	Fard	MeOH + chloroform (1:1)	7.5 ± 0.3
E1	Lulu	Acetone + water (1:1)	5 ± 0.5
E2	Lulu	Ethyl acetate	7.5 ± 0.3
F1	Mabroom	Acetone + water (1:1)	2.5 ± 0.5
F3	Mabroom	MeOH + chloroform (1:1)	5 ± 0.3
	Positive Control (Vancomycin)		10 µg/mL

Values are mean (*n* = 3) ± S.D. of three independent experiments.

## References

[1] Botes A., Zaid A. (2002). Date Production Support Program.

[2] Al-Farsi M.A., Lee C.Y. (2008). Nutritional and functional properties of dates: A review. Crit. Rev. Food Sci. Nutr..

[3] Biglari F., AlKarkhi A.F., Easa A.M. (2009). Cluster analysis of antioxidant compounds in dates (*Phoenix dactylifera*): Effect of long-term cold storage. Food Chem..

[4] Allaith A.A.A. (2008). Antioxidant activity of Bahraini date palm (*Phoenix dactylifera* L.) fruit of various cultivars. Int. J. Food Sci. Technol..

[5] Oni S.O., Adeosun A.M., Ladokun O.A., Ighodaro O.M., Oyedele M.O. (2015). Nutritional and phytochemical profile of Niger cultivated date palm (*Phoenix dactylifera* L.). J. Food Nutr. Sci..

[6] Eid N.M.S., Al-Awadi B., Vauzour D., Oruna-Concha M.J., Spencer J.P.E. (2013). Effect of cultivar type and ripening on the polyphenol content of date palm fruit. J. Agric. Food Chem..

[7] Saafi E.B., El Arem A., Issaoui M., Hammami M., Achour L. (2009). Phenolic content and antioxidant activity of four date palm (*Phoenix dactylifera* L.) fruit varieties grown in Tunisia. Int. J. Food Sci. Technol..

[8] Hong Y.J., Tomas-Barberan F.A., Kader A.A., Mitchell A.E. (2006). The flavonoid glycosides and procyanidin composition of deglet noor dates (*Phoenix dactylifera*). J. Agric. Food Chem..

[9] Maier V.P., Metzler D.M. (1965). Quantitative changes in date polyphenols and their relation to browning. J. Food Sci..

[10] Hammouda H., Chérif J.K., Trabelsi-Ayadi M., Baron A., Guyot S. (2013). Detailed polyphenol and tannin composition and its variability in Tunisian dates (*Phoenix dactylifera* L.) at different maturity stages. J. Agric. Food Chem..

[11] Roger R.C., Hadidane R., Biard J.F., Bouke F.K. (1987). High performance liquid and thin-layer chromatographic determination of phenolic acids in palm (*Phoenix dactylifera*) products. Food Chem..

[12] Lotito S.B., Frei B. (2006). Consumption of flavonoid-rich foods and increased plasma antioxidant capacity in humans: Cause, consequence, or epiphenomenon?. Free Radic. Biol. Med..

[13] Aldhaheri A., Alhadrami G., Aboalnaga N., Wasfi I., Elridi M. (2004). Chemical composition of date pits and reproductive hormonal status of rats fed date pits. Food Chem..

[14] Habib H.M., Ibrahim W.H. (2009). Nutritional quality evaluation of eighteen date pit varieties. Int. J. Food Sci. Nutr..

[15] Almana H.A., Mahmoud R.M. (1994). Palm date seeds as an alternative source of dietary fibre in Saudi bread. Ecol. Food Nutr..

[16] Hamada J.S., Hashim I.B., Sharif A.F. (2002). Preliminary analysis and potential uses of date pits in foods. Food Chem..

[17] Bhat R.S., Al-Daihan S. (2012). Antibacterial properties of different cultivars of *Phoenix dactylifera* L. and their corresponding protein content. Ann. Biol. Res..

[18] Perveen K., Bokhari N., Soliman D. (2012). Antibacterial activity of *Phoenix dactylifera* L. leaf and pit extracts against selected Gram negative and Gram positive pathogenic bacteria. J. Med. Plants Res..

[19] Yassin N.N. (2012). Antibacterial effect of date palm (*Phoenix dactylifera* L.) pit aqueous extract on some bacteria cause urinary tract infection. Diyala J. Pure Sci..

[20] Hamad I., Elgawad H.A., Al Jaoun S., Zinta G., Asard H., Hassan S., Hegab M., Hagagy N., Selim S. (2015). Metabolic analysis of various date palm fruit (*Phoenix dactylifera* L.) cultivars from Saudi Arabia to assess their nutritional quality. Molecules.

[21] Saleh E.A., Tawfik M.S., Abu-Tarboush H.M. (2011). Phenolic contents and antioxidant activity of various date palm (*Phoenix dactylifera* L.) fruits from Saudi Arabia. Food Nutr. Sci..

[22] AOAC (1995). Official Methods of Analysis.

[23] Hanson S.W.F., Olley J. (1963). Application of the Bligh and Dyer method of lipid extraction to tissue homogenates. Biochem. J..

[24] Minister of Agriculture, Fish & Food (1995). Manual of Nutrition.

[25] Singleton V.L., Rossi J.A. (1965). Colorimetry of total phenolics with phosphomolybdic–phosphotungstic acid reagents. Am. J. Enol. Vitic..

[26] Shui G., Leong L.P. (2006). Residue from star fruit as valuable source for functional food ingredients and antioxidant nutraceuticals. Food Chem..

[27] Kim D.O., Jeong S.W., Lee C.Y. (2003). Antioxidant capacity of phenolic phytochemicals from various cultivars of plums. Food Chem..

[28] Khan M.A., Inayat H., Khan H., Saeed M., Khan I., Rahman I. (2011). Antimicrobial activities of the whole plant of *Cestrum nocturnum* against pathogenic microorganisms. Afr. J. Microbiol. Res..

[29] Wikler M.A. Methods for Dilution Antimicrobial Susceptibility Test for Bacteria that Grow Aerobically. https://simpleshowoflove.weebly.com/uploads/1/4/0/7/14073276/agar_dilution_assay.pdf.

[30] Khalid S., Khalid N., Khan R.S., Ahmed H., Ahmad A. (2017). A review on chemistry and pharmacology of Ajwa date fruit and pit. Trends Food Sci. Technol..

[31] Al-Shahib W., Marshall R.J. (2003). Fatty acid content of the seeds from 14 varieties of date palm *Phoenix dactylifera* L.. Int. J. Food Sci. Technol..

[32] Ghnimi S., Umer S., Karim A., Kamal-Eldin A. (2017). Date fruit (*Phoenix dactylifera* L.): An underutilized food seeking industrial valorization. NFS J..

[33] Goni I., Valdivieso L., Garcia-Alonso A. (2000). Nori seaweed consumption modifies glycemic response in healthy volunteers. Nutr. Res..

[34] Guillon F., Champ M. (2000). Structural and physical properties of dietary fiber, and consequence of processing on human physiology. Food Res. Int..

[35] Mrabet A., Rodríguez-Gutiérrez G., Rodríguez-Arcos R., Guillén-Bejarano R., Ferchichi A., Sindic M., Jiménez-Araujo A. (2016). Quality characteristics and antioxidant properties of muffins enriched with date fruit (*Phoenix dactylifera* L.) fiber concentrates. J. Food Qual..

[36] Mrabet A., Rodríguez-Gutierrez G., Rubio-Senent F., Hamza F., Rodríguez-Arcos R., Guillen-Bejarano R., Sindic M., Jiménez-Araujo A. (2017). Enzymatic conversion of date fruit fiber concentrates into a new product enriched in antioxidant soluble fiber. LWT Food Sci. Technol..

[37] Otles S., Ozgoz S. (2014). Health effects of dietary fiber. Acta Sci. Pol. Technol. Aliment..

[38] Mansouri A., Embarek G., Kokkalou E., Kefalas P. (2005). Phenolic profile and antioxidant activity of the Algerian ripe date palm fruit (*Phoenix dactylifera*). Food Chem..

[39] Wu X., Beecher G., Holden J., Haytowitz D., Gebhardt S., Prior R. (2004). Lipophilic and hydrophilic antioxidant capacities of common foods in the United States. J. Agric. Food Chem..

[40] El Hadrami A., Daayf F., El Hadrami I., Jain S.M., Al-Khayri J.M., Johnson D.V. (2011). Secondary metabolites of date palm. Date Palm Biotechnology.

[41] Amira E., Behija S.E., Beligh M., Lamia L., Manel I., Mohamed H., Lotfi A. (2012). Effects of the ripening stage on phenolic profile, phytochemical composition and antioxidant activity of date palm fruit. J. Agric. Food Chem..

[42] Samad M.A., Hashim S.H., Simarani K., Yaacob J.S. (2016). Antibacterial properties and effects of fruit chilling and extract storage on antioxidant activity, total phenolic and anthocyanin content of four date palm (*Phoenix dactylifera*) cultivars. Molecules.

[43] Shakiba M., Kariminik A., Parsia P. (2011). Antimicrobial activity of different parts of *Phoenix dactylifera*. Int. J. Mol. Clin. Microbiol..

[44] Khatami M., Shahram P. (2015). *Phoenix dactylifera* (date palm) pit aqueous extract mediated novel route for synthesis high stable silver nanoparticles with high antifungal and antibacterial activity. IET Nanobiotechnol..

[45] Al-Daihan S., Bhat R.S. (2012). Antibacterial activities of extracts of leaf, fruit, seed and bark of *Phoenix dactylifera*. Afr. J. Biotechnol..

[46] Radfar R., Farhoodi M., Ghasemi I., Khaneghah A.M., Shahraz F., Hosseini H. (2019). Assessment of phenolic contents and antioxidant and antibacterial activities of extracts from four varieties of Iranian date palm (*Phoenix dactylifera* L.) seeds. Appl. Food Biotechnol..

[47] El Sohaimy S., Abdelwahab A., Brennan C., Aboul-enein A. (2015). Phenolic content, antioxidant and antimicrobial activities of Egyptian date palm (*Phoenix dactylifera* L.) fruits. Aust. J. Basic Appl. Sci..

[48] Belmir S., Boucherit K., Boucherit-Otmani Z., Belhachemi M.-H. (2016). Effect of aqueous extract of date palm fruit (*Phoenix dactylifera* L.) on therapeutic index of amphotericin B. Phytothérapie.

[49] Hussain M.I., Reigosa M.J. (2011). Allelochemical stress inhibits growth, leaf water relations, PSII photochemistry, non-photochemical fluorescence quenching and heat energy dissipation in three C3 perennial species. J. Exp. Bot..

[50] Hussain M.I., Reigosa M.J. (2014). Evaluation of herbicide potential of sesquiterpene lactone and flavonoid: Impact on germination, seedling growth indices and root length in *Arabidopsis thaliana*. Pak. J. Bot..

[51] Hussain M.I., Qamar-Abbas S., Reigosa M.J. (2017). Biological activities and novel applications of secondary metabolite coumarins. Planta Daninha.

[52] Dayan F.E., Cantrell C.L., Duke S.O. (2009). Natural products in crop protection. Bioorg. Med. Chem..

[53] Scalbert A., Williamson G. (2000). Dietary intake and bioavailability of polyphenols. J. Nutr..

[54] Cushnie T.T., Lamb A.J. (2005). Antimicrobial activity of flavonoids. Int. J. Antimicrob. Agents.

[55] Koffi E., Sea T., Dodehe Y., Soro S. (2010). Effect of solvent type on extraction of polyphenols from twenty three Ivorian plants. J. Anim. Plant Sci..

[56] Kallithraka S., Garciaviguera C., Bridle P., Bakker J. (1995). Survey of solvents for the extraction of grape seed phenolics. Phytochem. Anal..

[57] Yilmaz Y., Toledo R.T. (2006). Oxygen radical absorbance capacities of grape/wine industry byproducts and effect of solvent type on extraction of grape seed polyphenols. J. Food Compos. Anal..

[58] Kajdzanoska M., Petreska J., Stefova M. (2011). Comparison of different extraction solvent mixtures for characterization of phenolic compounds in strawberries. J. Agric. Food Chem..

